# De novo design of the global transcriptional factor Cra‐regulated promoters enables highly sensitive glycolysis flux biosensor for dynamic metabolic control

**DOI:** 10.1111/1751-7915.14166

**Published:** 2022-12-20

**Authors:** Yuan Zhu, Huaxiao Gao, Jian Zhang, Jingyu Zhao, Qingsheng Qi, Qian Wang

**Affiliations:** ^1^ National Glycoengineering Research Center, State Key Laboratory of Microbial Technology Shandong University Qingdao China

## Abstract

Glycolytic flux is a fundamental index in microbial cell factories. A glycolytic flux biosensor that can monitor glucose metabolism efficiency is a promising strategy in rewiring metabolic flux to balance growth and biosynthesis. A key design feature of the glycolytic flux biosensors is the interaction between the global transcriptional factor Cra and its regulated promoters. However, overexpression and mutation of Cra has unpredictable effects on global metabolism in *Escherichia coli*. Therefore, new orthogonal biosensor design strategies should be developed to circumvent metabolic issues. In this report, the promoters in glycolytic flux biosensor were replaced with synthetic promoters of varying strengths or phage‐derived promoters, and the Cra DNA‐binding sites were deployed into promoters at different positions and distances to yield biosensors. The de nova biosensors that depended on Cra could sense Fructose‐1,6‐diphosphate (FBP) with broad dynamic ranges and low basal leakage. Then the negative‐response biosensors were applied to fine‐tune the target ATP synthesis gene, leading to the desired increase in pyruvate production (the highest 9.66 g/L) and cell growth. Moreover, the membrane synthesis gene *plsC* was also dynamically activated by the positive‐response biosensor, leading to effective accumulation of lycopene in the cell membrane and a 50‐fold increase in lycopene titre (100.3 mg/L) when compared with the control strain, demonstrating the effective and broader usages of our biosensors.

## Graphical abstract

Glycolysis is the primary metabolic pathway in all living organisms. Maintaining the balance of glycolysis flux and biosynthetic pathways is the crucial matter involved in the microbial cell factory. The glycolysis flux biosensor based on global transcription factors Cra that can sense in vivo fructose‐1,6‐diphosphate (FBP) was a powerful tool for fine‐tuning and rewiring pathway flux. In order to avoid the influence on global metabolism generated by overexpression of global transcription factors, we designed and adjusted the high‐performance positive‐ and negative‐response biosensors using promoter do novo design for varied thresholds and dynamic ranges.

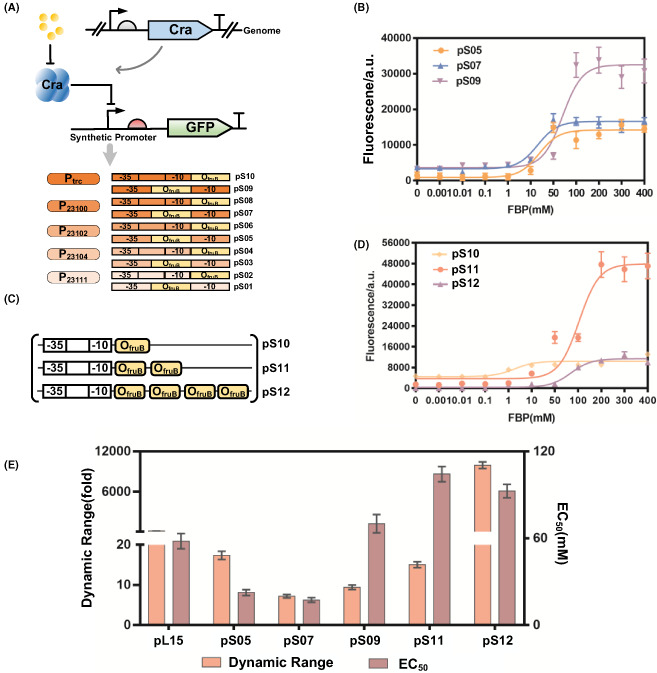

## INTRODUCTION

Metabolic engineering strategies enable the design of microbial cell factories to produce a variety of chemicals, pharmaceuticals, materials and functional nutraceuticals economically (Choi et al., 2019; Nielsen & Keasling, 2016). The static regulation strategies such as deletion, substitution or addition of necessary genes are usually required strategies to optimize metabolic fluxes and ultimately processes. These traditional strategies may occasionally lead to metabolic imbalances and the accumulation of intermediates. Thus, further design is typically required because of the complexity of metabolism and its regulation (English et al., 2021; Moser et al., 2018). As an alternative, dynamic regulation is a promising strategy for fine‐tuning metabolic fluxes in microbial cell factories (Moser et al., 2018). Genetically encoded biosensors are a key component of dynamic metabolic control (Liu et al., 2015). Biosensor‐based genetic circuits have been developed for autonomous control of metabolic fluxes by responding to intracellular metabolites, such as fatty acyl‐CoA (Zhang et al., 2012), malonyl‐CoA (Xu et al., 2014), pyruvate (Xu et al., 2020), quorum signal molecules acyl‐homoserine lactones (Gupta et al., 2017) and other stimuli. Fructose‐1,6‐diphosphate (FBP) is a key intermediate metabolite that links the upper and lower reaction steps of the glycolytic pathway and has been shown to reflect glycolysis flux (Kochanowski et al., 2013). FBP levels can be sensed by the global bifunctional transcription regulator Cra to regulate gene expression associated with glycolysis and gluconeogenesis pathways (Chin et al., 1989; Ishihama, 2010; Leclerc et al., 1990). We previously created a set of programmable and bifunctional glycolysis flux biosensors to construct FBP‐responsive genetic circuits for dynamic dual control (activation and inhibition) of glycolysis flux in *Escherichia coli*. These engineered glycolysis flux biosensors were used to fine‐tune the glycolysis flux to balance the biosynthesis of mevalonate and N‐acetylglucosamine effectively, with the highest mevalonate titre of 111.3 g/L achieved in a 1 L fermenter (Zhu et al., 2021).

Currently, dynamic metabolic control driven by biosensor‐based genetic circuits is usually performed using specific transcription factors (TFs), which have unique advantages in biosensor design (Kim et al., 2020). In contrast, global TFs (Deng et al., 2022) can activate or repress the co‐expression of multiple genes simultaneously in several specific pathways. The multi‐targets and multiplex characteristics (Mannan et al., 2017) of global TFs make them more extensive and flexible in dynamic regulation applications. However, the use of global TFs presents several challenges. The design of TF‐based biosensors often requires overexpression of TFs (Merulla & van der Meer, 2016). Global TF overexpression can affect multiple targeted regulatory genes, thereby affecting overall cellular metabolism because global TFs are usually endogenous or homologues in the host. Thus, strategies targeting the overexpression of global TFs should be avoided for tuning biosensor functions (Zhang et al., 2021).

Promoters are fundamental elements and the first gate of gene regulation, and screening promoters with excellent performance remains an essential consideration in whole‐cell biosensor design. Conventional promoter engineering approaches have focused on directed evolution for high‐throughput screening of optimal promoters (Li et al., 2015). Prokaryotic promoter engineering usually targets two core elements, including the RNA polymerase‐binding sites (−10 and −35 sites) that determine promoter activity and transcription factor‐binding sites (TFBSs), which control gene expression (Chen et al., 2018). The de novo design and optimization of promoters based on the bottom‐up concept have not been explored in detail.

In this report, to avoid the overexpression of the global TF Cra, we focused on the de novo design and optimization of Cra‐regulated promoters to tune the function of glycolytic flux biosensors. De novo design of active‐ and negative‐response Cra‐regulated promoters was achieved by replacing native promoters with a series of different strength synthetic promoters or with a phage‐derived promoter (Liu et al., 2019) for strict regulation. De novo design also involved embedding TFBSs of different activation and inhibitory effects within the above synthetic promoters and deploying TFBSs at different positions in the promoter. After optimization, positive‐ and negative‐response promoters with low leakage and high fold changes were selected and successfully used in the dynamic regulation of pyruvate and lycopene biosynthesis.

## EXPERIMENTAL PROCEDURES

### Strains and construction of plasmids

All strains and plasmids used in this study are summarized in Tables S1 and S2, respectively. Molecular cloning and manipulation of plasmids were performed using *E. coli* DH5α cells. All oligonucleotides used are listed in Table S3.

High‐fidelity DNA polymerase (TaKaRa, Japan) was used for plasmid construction, and 2× Taq MasterMix (CWBIO, China) was used for the colony polymerase chain reaction (PCR). Strain DH5α was transformed with the assembled plasmid, and colony PCR and sequencing were performed to verify the correctness of the plasmid. pS01–pS15 were obtained by transforming the large fragment after treatment with T4 PNK and T4 ligase, which replaced the *gfp* promoter of pL09 with 15 modified promoters using the primers PSM1‐F1/PSM1‐F2/PS‐R, PSM2‐F1/PSM2‐F2/PS‐R, PSM3‐F1/PSM3‐F2/PS‐R, PSM4‐F1/PSM4‐F2/PS‐R, PSM5‐F1/PSM5‐F2/PS‐R, PSM6‐F1/PSM6‐F2/PS‐R, PSM7‐F1/PSM7‐F2/PS‐R, PSM8‐F1/PSM8‐F2/PS‐R, PSM9‐F1/PSM9‐F2/PS‐R, PSM10‐F1/PSM10‐F2/PS‐R, PSM11‐F1/PSM11‐F2/PS‐R, PSM12‐F1/PSM12‐F2/PS‐R, PSM13‐F1/PSM13‐F2/PS‐R, PSM14‐F1/PSM14‐F2/PS‐R, PSM15‐F1/PSM15‐F2/PS‐R. Strains S01–S15 were obtained by transforming the plasmids pS01–pS15 into *E. coli* strain BW‐*pfkA*. All experiments related to gene knockout and the replacement of the promoter on the genome were performed using the homologous recombination method. The ability of the biosensors to detect glycolysis was tested by replacing the promoters of *pfkA* genes on the genome with four promoters screened in the IGEM library using homologous recombination. The starting strain of pyruvate fermentation was the *E. coli* BW25113 with the *ackA‐ptA‐poxB* gene knocked out, This strain was named BW. *crtE*, *crtB* and *crtI* were cloned into the plasmid Ptrc99A to obtain the plasmid pEBI. *mk*, *pmk*, *pmvd* and *idi* were cloned into the plasmid PCDFduet to obtain the plasmid pMKD.

### Cultivation conditions

*Escherichia coli* DH5α cells were used for cloning purposes and propagated in Luria‐Bertani (LB) medium (5 g/L yeast extract, 10 g/L tryptone and 10 g/L NaCl) at 37°C under aeration. M9 medium (15.6 g/L Na_2_HPO_4_·12H_2_O, 3 g/L KH_2_PO_4_, 1 g/L NaCl, 1 g/L NH_4_Cl, 0.494 g/L MgSO_4_·7H_2_O, 0.0153 g/L CaCl_2_.H_2_O, 0.01 g/L FeSO_4_·7H_2_O and 2 g/L yeast extract, with vitamin B1 added to a final concentration of 10 μg/ml) was used for the characterization of the biosensor. Strains were cultivated in LB medium and then transferred to M9 medium when characterizing a biosensor. For pyruvate fermentation, 50‐ml shake flask cultures were started with 2% inoculation from a 5‐ml LB culture, and then the strains were fermented in an M9 medium (without vitamin B1) at 37°C and 220 rpm. Antibiotics were added when necessary (100 μg/ml ampicillin, 50 μg/ml kanamycin, 17 μg/ml chloramphenicol and 100 μg/ml spectinomycin). Glucose (30 g/L) was supplied when the glucose level was below 10 g/L. For lycopene production, 50‐ml shake flask cultures were also started by 2% inoculation from a 5‐ml LB culture. The 50‐ml cultures contained lycopene fermentation medium containing 30 g/L glucose and were shaken at 37°C in a rotary shaker (220 rpm) for 72 h. The lycopene fermentation medium used for lycopene production in bioreactors contained 16 g/L yeast extract, 24 g/L tryptone and 5 g/L NaCl.

### Fluorescence measurements for biosensor characterization

The fluorescence intensity of the glycolysis flux biosensor promoters was detected by transforming *E. coli* strain BW‐*pfkA* with each biosensor plasmid separately. All molecular biological manipulations were performed as described previously (Zhang et al., 2020). Single colonies were then picked and inoculated into LB medium supplied with 100 μg/ml ampicillin and grown at 37°C and 220 rpm. After 12 h, the seed cultures were collected, washed, and 200 μl of fresh M9 medium containing 10 g/L acetate and 100 μg/ml ampicillin was inoculated with 2% (v/v) overnight seed culture in a 96‐well fluorescence plate and grown at 37°C and 500 rpm. Different concentrations of FBP, 0.001, 0.01, 0.1, 1, 5, 10, 50, 100, 200, 300 and 400 mM, were added to the cell culture to regulate the expression of the reporter gene, which was controlled by the biosensor promoter. The optical density (OD; 600 nm) and GFP fluorescence (excitation, 488 nm; emission, 520 nm) were detected simultaneously using a Cytation microplate reader (BioTek). All experiments were performed in triplicate.

### Analytical methods

The OD was measured at 600 nm with a spectrophotometer (Shimazu, Japan). For pyruvate fermentation, fermentation samples were centrifuged at 10,000 *g* for 5 min, and the supernatant was used for extracellular metabolite detection. Glucose, acetate, lactate and pyruvate were quantitatively determined using an HPLC system (Shimadzu, Japan) equipped with a refractive index detector (RID‐10A; Shimadzu) and an Aminex HPX‐87H ion exclusion column (Bio‐Rad, USA), as described previously (Zhu et al., 2021). One‐millilitre aliquots of the fermentation broth were centrifuged to collect the cells and to analyse the lycopene contents. The cells were resuspended in 0.75 ml dimethyl sulphoxide and incubated for 10 min at 55°C and then for 15 min at 50°C after adding an equal volume of acetone. The samples were centrifuged (10,000 *g* for 5 min). The supernatants containing lycopene or other carotenoids were analysed using the Shimadzu LC‐20AT HPLC system equipped with a variable‐wavelength detector (475 nm) and an Agilent ZORBAX Eclipase XDB‐C18 column. The mobile phase (at 30°C) consisted of 1 ml/min methanol, acetonitrile and dichloromethane (42:42:16).

### Quantitative real‐time PCR

The primers used are listed in Table S3. The sequence of 16 s rRNA was used as the internal control. The messenger RNA (mRNA) level was measured through quantitative real‐time PCR. Samples for extracting mRNA were harvested and frozen immediately at −80°C. Total mRNA was extracted using the RNeasy Mini Kit (Tiangen). cDNA was obtained through reverse transcription, and quantitative real‐time PCR was performed in a 96‐well plate with a total reaction volume of 20 μl per well in QuantStudioTM3 (ThermoFisher) using SYBR Premix Ex Taq II (Perfect Real Time), according to the manufacturer's instructions (TaKaRa).

## RESULTS

### Performance improvement of the glycolysis flux biosensors by promoter design

**FIGURE 1:**
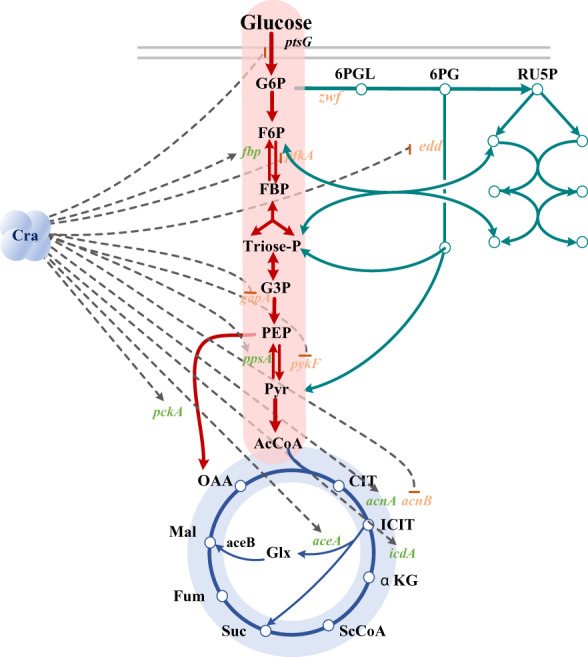
Global regulation of the central carbon metabolic pathway by the transcription factor Cra.

Cra inhibits glycolysis by targeting *pykF*, *pfkA*, *edd* and *eda* and activates gluconeogenesis through targeting *fbp*, *ppsA* and so on (Cozzone & El‐Mansi, 2005; Ramseier et al., 1995; Sarkar et al., 2008; Figure 1). The Cra could be used to control metabolic flux by inhibiting or activating genes expression. Previously, we used Cra to regulate the promoters P_*ppsA*_ and P_*fruB*_ and their Cra DNA‐binding sites (DBS) in the design and construction of a series of negative‐response biosensors and positive‐response biosensors, respectively. Only two positive‐response biosensors, pL09 and pL10, harbouring P_23107_ with O_*fruB*_ between the −35 and − 10 regions and downstream of the −10 region, showed improved dynamic range with 1.41‐ and 1.46‐fold dynamic ranges, respectively (Zhu et al., 2021). In this previous study, the Cra gene overexpressed to improve biosensor function. To avoid global transcription and metabolic changes in Cra‐regulated promoters caused by overexpression of Cra, we focused on engineering de novo promoters for tuning the function of the biosensor and obtaining more practical high‐performance biosensors.

**FIGURE 2:**
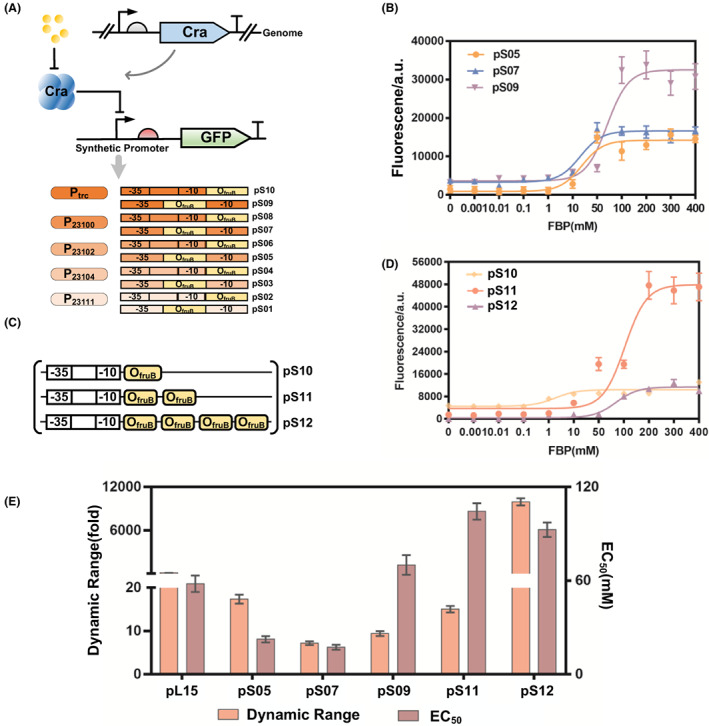
De novo promoter engineering design of positive‐response glycolysis flux biosensors. (A) The TFBS O_fruB_ was placed between the −35 and −10 regions and downstream of the −10 region of the promoters P_23111_, P_23104_, P_23102_, P_23100_ and P_trc_, respectively, to obtain the positive‐response biosensors pS01–pS10. (B) Characterization of biosensors pS05, pS07 and pS09, which have higher dynamic range and lower basal leaky expression. The lines represent Hill function fits to the data. (C) Positive‐response biosensors were obtained by connecting different numbers of O_fruB_ TFBSs. (D) Characterization of biosensors pS10, pS11 and pS12. (E) Dynamic range and EC_50_ of the positive‐response biosensors pL15 (pL15 is a positive‐response biosensor with optimal parameters constructed the previous study) pS05, pS07, pS09, pS11 and pS12. The dynamic range mentioned in this study is multiples of the maximum and minimum fluorescence values, and the value of EC_50_ is the substrate concentration at half of the fluorescence value.

Initially, five different promoters with different strengths, P_23111_, P_23104_, P_23102_, P_23100_ and P_trc_ (the sequences of these promoters are listed in Table S4), were selected as the core region of the promoter. Additionally, the Cra DBS on the *fruB* promoter, O_*fruB*_, was placed between the −35 and −10 regions and downstream of the −10 region of the five promoters, respectively, to obtain the artificially activated promoters (Figure 2A). All Cra‐regulated promoters were positioned before *gfp* to obtain plasmids pS01–pS10. Biosensor cells were cultured in an M9 medium with sodium acetate as the carbon source to eliminate the effect of in vivo FBP. The FBP dose–response (0–400 mM) curves revealed that biosensors pS05, pS07 and pS09 yielded the highest dynamic range and lowest basal leakage expression (Figure 2B). These plasmids also exhibited left‐shifted response thresholds of 22.4, 17.35 and 70 mM, respectively (Figure 2E). The results showed that as the strength of the core promoters increased (from P_23102_ to P_trc_; Figure S1), the maximum fluorescence value of the responsive biosensors they constituted increased. Altering the transcriptional efficiency scales vertically with the response curve. Moreover, the repression efficiency was strongest when the position of the TFBS O_*fruB*_ was located between −10 and −35 sites (~18 bp spacer), which is consistent with previous studies (Liu et al., 2019; Lutz & Bujard, 1997).

However, although the dynamic range and maximum output of the response curves increased, the leakage expression of these biosensors with strong promoters was higher than those with weak promoters. To obtain positive‐response biosensors with large dynamic ranges and low leakage expression, we incorporated two and four Cra DBS O_*fruB*_ in series downstream of the −10 region of promoter P_trc_ to obtain biosensors pS11 and pS12, respectively (Figure 2C). Characterization of these biosensors revealed that the biosensor containing two repeats of O_*fruB*_ had a lower leaky expression, and pS12 with four O_*fruB*_ had almost no leaky expression (Figure 2D). Multiple Cra DBS may increase the resistance to RNA polymerase escape from the promoter, thus reducing basal leakage and therefore increasing the dynamic range. Interestingly, the absolute maximum fluorescence output also increased. The incorporation of DBS may improve the promoter strength. Here, positive‐response biosensors with larger dynamic range, lower threshold were obtained through the engineering design of the promoter compared with previously constructed positive‐response biosensor pL15 (the dynamic range of pL15 is 148‐fold and the EC_50_ is 58 mM).

**FIGURE 3:**
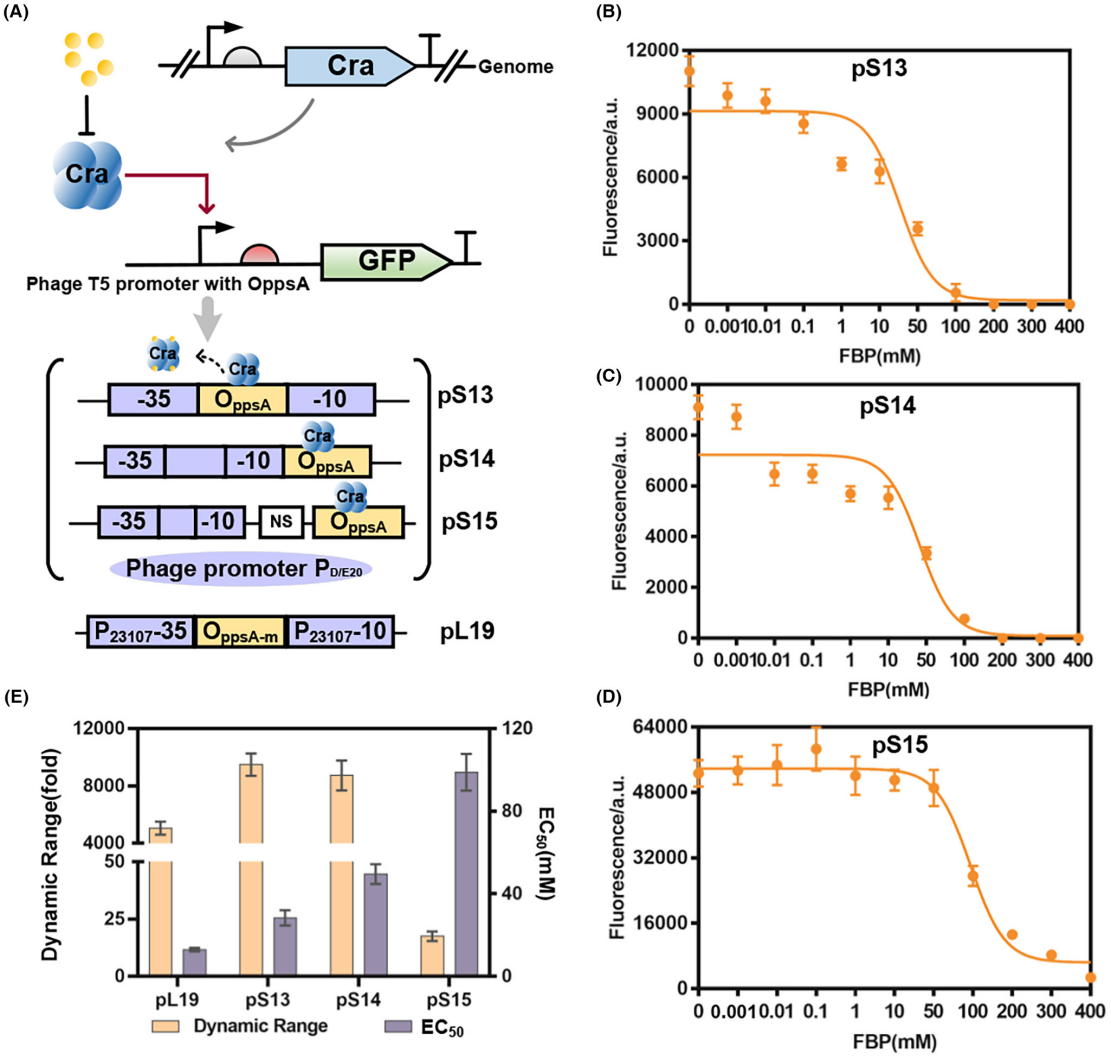
Engineering of negative‐response glycolysis flux biosensors. (A) The design of negative‐response biosensors pS13, pS14 and pS15 harnessing the *Escherichia coli* phage T5 promoter; the design of negative‐response biosensor pL19. Characterization of the negative‐biosensors pS13 (B), pS14 (C) and pS15 (D). “NS” refers to nonsense sequence, which is the same length as O_ppsA_, The lines represent Hill function fits to the data. (E) The dynamic range and EC_50_ of the negative‐biosensors pS13, pS14 and pS15and pL19 (pL19 is a negative‐response biosensor with the best parameters constructed in a previous study).

As for negative‐response biosensors, we previously screened Cra DBS O_*ppsA*_, which has a strong repression effect. Here, a series of strategies based on engineering the biosensor promoter was also adopted to increase the dynamic range. *E. coli* phage T5 promoters are an orthogonal and highly repressible bacterial expression suite, with basal transcription levels falling below those of the most tightly known bacterial promoters (Ruegg et al., 2018). Therefore, we embedded the operator O_*ppsA*_ between the −35 and −10 regions and downstream of the −10 regions of immediate‐early *E. coli*‐phage T5 promoter P_D/E20_ to yield new negative‐response biosensors pS13 and pS14 (Figure 3A). The fluorescence signal of biosensors harbouring P_D/E20_ engineered promoters pS13 and pS14 increased up to 9729‐fold and 8719‐fold upon induction, enabling a larger dynamic range and very low leaky expression when compared with those obtained by existing bacterial expression systems (Figure 3B,C), demonstrating the advantage of our biosensors with bacteriophage‐derived promoters controlled by O_*ppsA*_. The pS13 and pS14 biosensors also exhibited left‐shifted response thresholds of 28.36 and 48.8 mM, respectively (Figure 3E). We adjusted the distance between the O_*ppsA*_ and the promoter to further enhance the absolute fluorescence value of the biosensors. O_*ppsA*_ was placed 16 bp [“NS” (nonsense sequence)] away from the promoter P_D/E20_, constructed the new promoter which regulated by Cra, named Pun, to obtain the biosensor pS15 (Figure 3D). The maximum output fluorescence value of the negative‐response biosensor increased to 48,000, which is higher than the values reported for pS13, pS14 and pL19 (pL19 is a negative‐response biosensor successfully constructed in a previous study, the dynamic range of pL19 is 5051‐fold and the EC_50_ is 12.95 mM). The molecular mechanism by which Cra can activate transcription in the negative‐response biosensors based on the O_ppsA_‐binding site and promoter P_D/E20_ is at present unknown and deserves further investigation.

### Detection of the intracellular glycolysis flux

**FIGURE 4:**
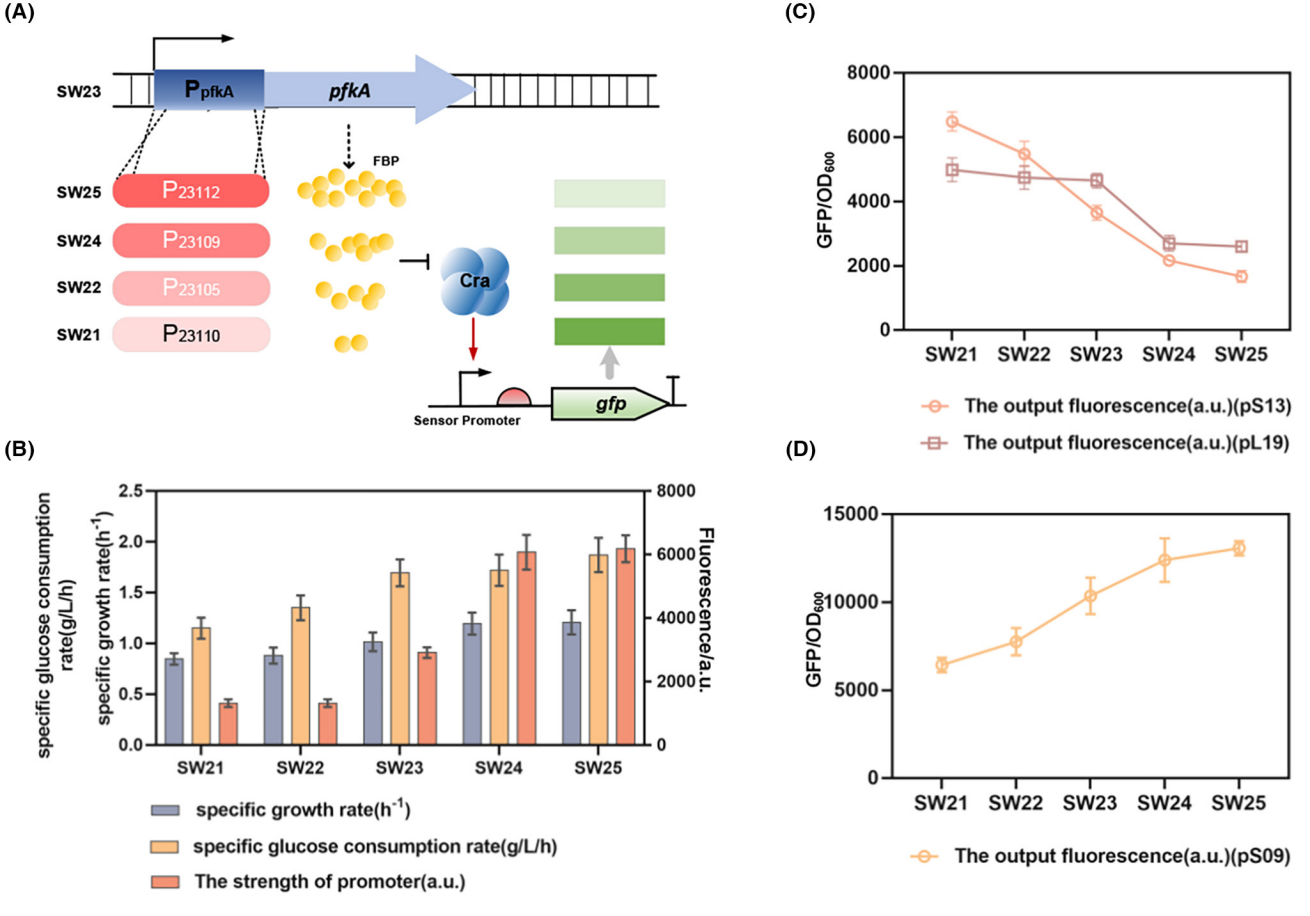
Monitoring glycolysis flux using newly constructed biosensors. (A) Construction of strains with different glycolysis fluxes. The strain with the promoter of *pfkA* replaced by the promoter P_23110_ is named SW21; the strain with the promoter of *pfkA* replaced by the promoter P_23105_ is named SW22; the strain with the promoter of *pfkA* replaced by the promoter P_23109_ is named SW24; the strain with the promoter of *pfkA* replaced by the promoter P_23112_ is named SW25; the wild strain is named SW23. (B) Characterization of the strength of promoters P23110, P23105, P*pfkA*, P23109 and P23112, and the specific growth rate and specific glucose consumption of strains SW21–SW25. (C) The negative‐response biosensors pL19 and pS13 were used to detect the glycolysis flux of the designed strains. (D) The positive‐response biosensor was used to detect the glycolysis flux of the designed strains.

In most cases, engineered strains may have minimal differences in glycolytic flux. Thus, glycolysis flux biosensors should be designed to be sensitive to slight differences in metabolic flux. *pfkA* is considered to be the key gene of glycolysis in *E. coli*. To verify this, we obtained a series of modified strains with different glycolytic fluxes by replacing the *pfkA* promoter on the genome with four promoters, P_23110_, P_23105_, P_23109_ and P_23112_ (Table S4), with varying strengths, naming SW21, SW22, SW24 and SW25 (Figure 4A). To compare the strength of these promoters, P_*pfkA*_ and the four promoters were, respectively, fused to *gfp* and introduced into plasmid pTrc99a to give pS16–pS20. The results of testing these promoters revealed that the fluorescence intensity ranking was P_23112_ > P_23109_ > P_*pfkA*_ > P_23105_ > P_23110_ (Figure 4B). The glycolytic flux of these four strains and the control strain BW25113 (SW23) were measured by the negative‐response biosensor pS13 with high dynamic range and a previously constructed negative‐response biosensor pL19. The results showed that both the negative‐response biosensor pS13 and pL19 can detect the glycolysis flux of these five strains. The fluorescence value of the five strains decreased in the following order SW21 > SW22 > SW23 > SW24 > SW25 (Figure 4C), indicating the glycolytic flux of these strains is SW25 > SW24 > SW23 > SW22 > SW21. At the same time, these five strains SW21‐SW25 were measured by the positive‐response biosensor pS09, the result shows that the fluorescence valve increasing with the increasing glycolysis flux (Figure 4D). to verify the accuracy of this biosensor in detecting glycolysis flux, the specific growth rate and the specific glucose consumption rate of SW21–SW25 were detected (Figure 4B). SW25 had the highest specific growth rate and specific glucose consumption rate, whereas SW21 had the lowest specific growth rate and specific glucose consumption rate. The output fluorescence intensity of the modified strains shows a negative correlation with the specific growth rate and specific glucose consumption rate. Compared with pL19, the biosensor pS13 is more sensitive to glycolysis flux detection (Figure 4C), which is more suitable for detecting strains with small flux differences. This result verified the accuracy of our constructed biosensors in detecting glycolytic flux, and the negative‐response biosensor pS13 showed the highest sensitivity and high dynamic range in detecting modified strains with minor differences in glycolytic flux, thus expanding the application scope of this biosensor.

### Dynamic control of ATP synthesis in pyruvate production

**FIGURE 5:**
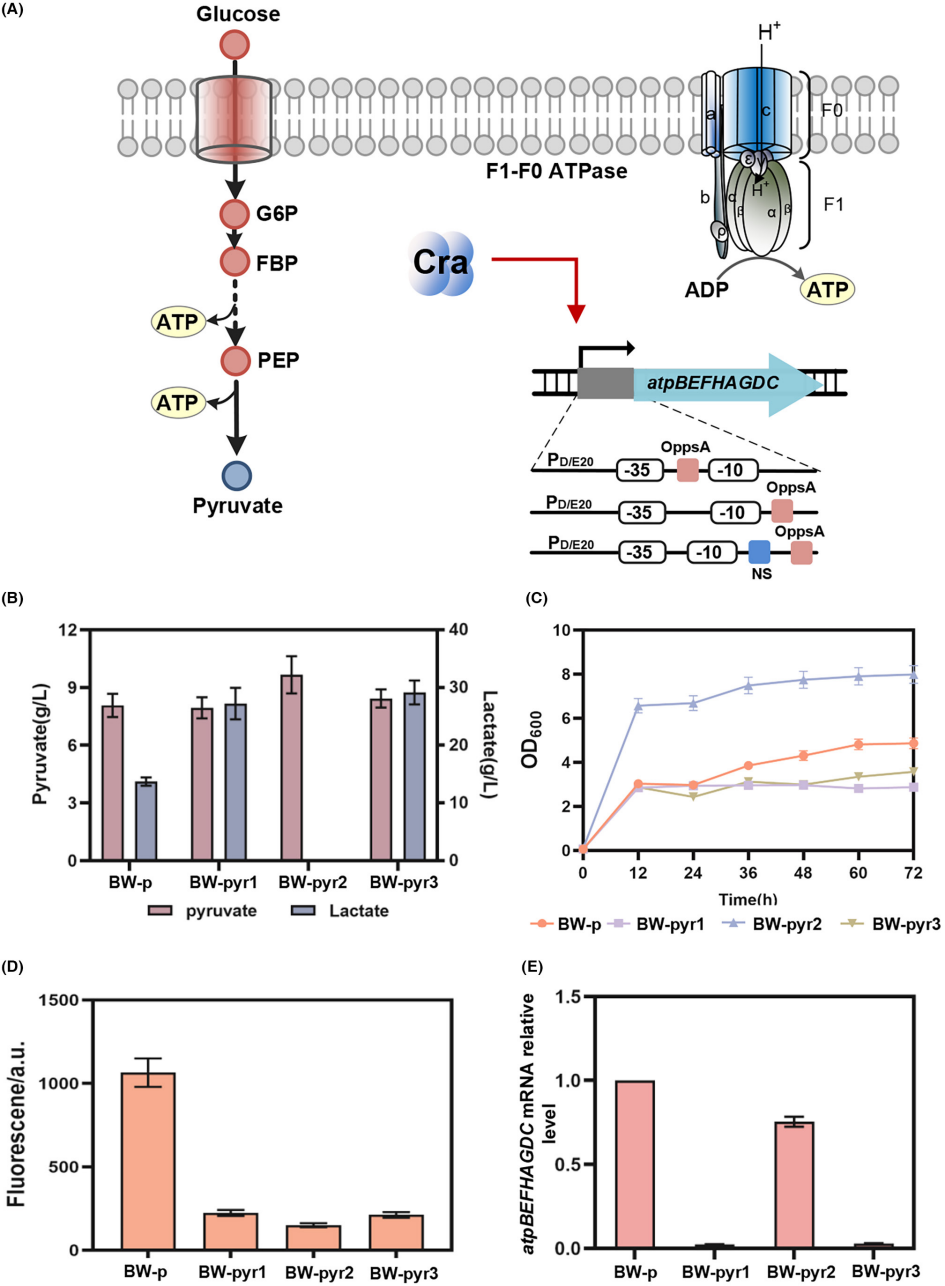
Application of the flux biosensor in pyruvate biosynthesis for balancing the ATP level. (A) The gene *atpBEFHAGDC* was regulated by negative‐response biosensors pS13, pS14 and pS15 (B) The accumulation of pyruvate and lactate of BW‐p, BW‐pyr1, BW‐pyr2 and BW‐pyr3. (C) The growth of strains BW‐p, BW‐pyr1, BW‐pyr2 and BW‐pyr3. (D) The glycolysis flux of strains BW‐p, BW‐pyr1, BW‐pyr2 and BW‐pyr3 was detected by pS13. (E) The relative expression level of *atpBEFHAGDC* by strains BW‐p, BW‐pyr1, BW‐pyr2 and BW‐pyr3.

Pyruvate is a key intermediate metabolite that plays an important role in glycolysis, gluconeogenesis, and amino acid and fatty acid metabolism. The demand for pyruvate is increasing with the continuing development of the biomedical industry (Akita et al., 2016). Pyruvate fermentation via glycolysis generates two molecules of ATP. Excess ATP represses the glycolysis flux by feedback inhibition of pyruvate kinase, representing the biggest bottleneck for pyruvate production (Koebmann et al., 2002). Here, we use the glycolysis flux biosensor to sense the glycolysis flux and control the ATP energy level, alleviating ATP feedback inhibition of pyruvate kinase. The starting strain was *E. coli* BW25113 with deletions of *ackA*, *pta* and *poxB* to eliminate acetate accumulation, named BW‐p. The ATP synthase genes *atpBEFHAGDC* involved in the synthesis and decomposition of ATP were dynamically downregulated using negative‐response biosensors to relieve ATP inhibition during pyruvate accumulation. The promoter P_*atp*_ of the ATP synthase genes on the genome was replaced with three negative‐response promoters, P_stm_ (pS13), P_str_ (pS14) and P_un_ (pS15; Figure 5A), to yield strains BW‐pyr1, BW‐pyr2 and BW‐pyr3 on the basis of strain BW‐p. (Figure 5A). After pyruvate fermentation, all strains showed different growth and physiological characteristics. The highest cell growth and pyruvate level, 9.66 g/L, was observed for BW‐pyr2 (Figure 5B,C), whereas control strain BW‐p accumulated 7.98 g/L pyruvate (Figure 5B). Surprisingly, no lactate was generated in BW‐pyr2. BW‐p, BW‐pyr1 and BW‐pyr3 accumulated 13.9, 27.22 and 29.81 g/L lactate, respectively (Figure 5B). The negative‐response biosensor pS13 was used to detect the glycolytic flux of BW‐p, BW‐pyr1, BW‐pyr2 and BW‐pyr3, the results showed that the glycolytic flux of the three strains with dynamic inhibition of ATP synthase gene increased compared with the control strain BW‐p (Figure 5D). The mRNA level of the ATP synthase gene in BW‐pyr1, BW‐pyr2 and BW‐pyr3 strains was inhibited by 90%, 25% and 90%, respectively, when compared with BW‐p (Figure 5E), verifying the different transcriptional inhibition effects of the negative‐response biosensors on ATP synthase. ATP levels were dynamically downregulated as the concentration of FBP increased to a certain threshold.

A decrease in cellular levels of ATP has previously been reported to increase fermented product yield and productivity (Boecker et al., 2021; Wada et al., 2007). In the pyruvate‐producing strain, dynamic inhibition of ATP synthesis decreased the cellular energy level, thus enhancing the glucose metabolism of this dynamically controlled strain. Enhancing glucose metabolism should improve fermentative production. Moreover, different inhibition levels of ATP resulted in different effects on metabolism and growth. This result also showed that ATPase should be inhibited to a moderate expression level to ensure a balance between growth and reducing power. Excessive ATP inhibition caused the production of large amounts of lactic acid from pyruvate even under aerobic conditions. Mild ATP inhibition gave increased pyruvate production and higher biomass accumulation.

### Dynamic control of cell membrane morphology for improved lycopene production

**FIGURE 6:**
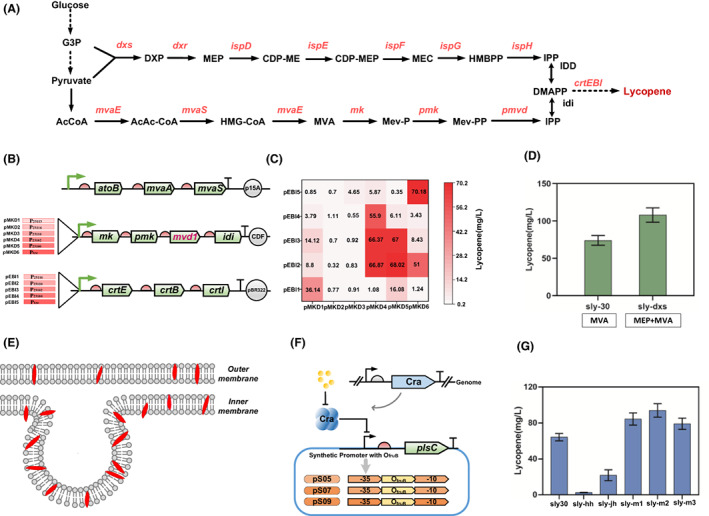
Dynamic control of membrane synthesis gene *plsC* by the glycolysis flux biosensor for improved lycopene production. (A) The production pathway of lycopene. (B) The genes that produce lycopene are expressed on different plasmids. (C) The production of strains sly01–sly30. (D) The production of lycopene with the pathway of MVA and both MVA and MEP. (E) Lycopene accumulates on the membrane. (F) The gene *plsC* was regulated by the active‐response biosensors pS05, pS07 and pS09. (G) Lycopene production dynamically regulates the expression of *plsC*.

Lycopene is an effective antioxidant used widely in pharmaceutical, nutrition and cosmetics fields (Harada, 2021). The biosynthesis of lycopene by *E. coli* includes the endogenous MEP pathway (Sun et al., 2014) and the exogenous MVA pathway (Figure 6A; Yang et al., 2016). Because lycopene is a hydrophobic terpene product that inserts into and accumulates in the cell membrane, cell membrane biosynthesis and its morphology indirectly affect the lycopene storage capacity and, therefore, accumulation of lycopene. Membrane engineering is a feasible strategy for increasing the production and accumulation of lycopene (Wu et al., 2018). Overexpression of diglyceride‐3‐phosphate synthesis pathway genes was reported to increase the amount of glycerophospholipids in *E. coli* cells (Janssen & Steinbuchel, 2014). However, overexpression of membrane proteins causes serious toxicity to the host and affects cell growth and product accumulation (Michou et al., 2020; Nannenga & Baneyx, 2011). Thus, to address this issue, we dynamically controlled the key gene related to the membrane‐synthesis pathway in lycopene‐producing *E. coli*.

The MVA pathway was then introduced by expressing *atoB*, *mvaS* and *mvaA* under the control of P_trc_ on plasmid p15A to obtain the plasmid pMVA. Genes *mk*, *pmk*, *idi* and *pmvd* were constructed on pCDFDuet‐1 to obtain pMKD. Genes *crtE*, *crtB* and *crtI* were constructed on pTrc99A to obtain pEBI (Figure 6B). The promoter intensities on pMKD and pEBI were then optimized by replacing six promoters with the different intensity promoters P23113, P23116, P23110, P23102, P23100 and Ptrc, and the appropriate expression intensity was determined by screening. pEBI with five different promoters—P23116, P23110, P23102, P23100 and Ptrc—was used to obtain plasmids pEBI1–pEBI5, and pMKD with six different promoters—P23113, P23116, P23110, P23102, P23100 and Ptrc—was used to generate plasmids pMKD1–pMKD6 (Figure 6B). These plasmids were combined successively and transformed into *E. coli* strain DH5α together with the plasmid pMVA. Engineered *E. coli* strains with two different plasmid combinations were named sly01–sly30. The fermentation results showed that gene expression intensities on pMKD and pEBI were strong, with the lycopene yield reaching a maximum of 70.13 mg/L (Figure 6C). Thus, pEBI5 and pMKD6 were selected for the following lycopene production experiment. Synergistic enhancement expression of the *dxs* gene from the MEP pathway further improved lycopene production to 100.8 mg/L (Figure 6D).

*plsC* is involved in the membrane‐synthesis pathway and can increase the supply of membrane lactone to increase the production of lycopene (Figure 6E). The potential metabolic burden of the *plsC* gene product was dynamically regulated by the positive‐response biosensor promoters pS05, pS07 and pS09 to give plasmids pLymc1, pLymc2and pLymc3, respectively (Figure 6F). These three plasmids were transferred into *E. coli* strain DH5α harbouring pEBI5 and pMKD6 for lycopene fermentation, named sly‐m1, sly‐m2 and sly‐m3, respectively. The strains slyhh and slyjh constitutively expressing *plsC* under a P_tac_ and P_23102_ promoter on plasmid pMVA, respectively, were used as controls. The results showed that dynamic control of *plsC* increased lycopene production by 30% when compared with the lycopene‐producing strain without *plsC* overexpression. Constitutive overexpression of *plsC* with the promoter P_tac_ only produced 2 mg/L lycopene, which is 0.02‐fold that of the dynamically controlled strain (Figure 6G). In the dynamical control strain, when cells enter into a vigorous growth period, FBP reaches a certain level, and *plsC* is activated to accelerate cell membrane formation. We have demonstrated that the positive‐response biosensors can be applied to regulate the production of metabolites in growing bacteria.

## DISCUSSION

Intracellular metabolite biosensors based on TFs have been used widely in high‐throughput screening and metabolic regulation. However, TFs do not always bind effectively to ligands, bind DNA tightly, or frequently recruit RNA polymerase. As a result, biosensors designed using these TFs have impaired sensing ability, low sensitivity, low dynamic range and narrow substrates over the linear detection range. Therefore, tuning biosensors is essential for dynamic control systems to function under industrial conditions appropriately.

Transcription factor engineering is a useful strategy to modify ligand specificity or change the affinity between small molecules and TFs to obtain high‐performance biosensors that have been proven previously. However, as modification of regulatory proteins may affect the global metabolism of bacteria, we have focused on the promoter design of glycolysis flux biosensors in this study. Phage‐derived promoters have greater sensitivity and responsiveness, thus giving biosensors with a high dynamic range. Harnessing the strength of core promoters increases the dynamic range, and increasing the number of DBS in series can reduce basal leakage. Finally, biosensor pS11 has no leaky expression, high sensitivity and a large dynamic range.

We have previously used constructed glycolysis flux biosensors to detect glycolysis fluxes in *E. coli* strains with different gene knockouts in glycolysis. Here, as the constructed negative‐response biosensor pS13 showed high sensitivity than previous constructed biosensors, it has an advantage over previously constructed biosensors when detecting strains with similar glycolysis fluxes.

The primary design function for dynamic control is to balance metabolic flux between cell growth and chemical production (Soma & Hanai, 2015) or avoid toxic intermediate accumulation by inhibiting an upstream pathway and activating a downstream pathway (Zhang et al., 2012). Several studies have changed the glycolysis flux by directly upregulating or suppressing key genes in the glycolysis pathway to control the glycolysis flux. Reasons for the unsuccessful attempts to increase glycolysis flux by overexpression of glycolysis enzyme have been presented (Koebmann et al., 2002). Herein, the first example was the dynamic downregulation of ATP synthesis and thus the production of excess ATP when the glycolytic flux reached a certain level. Such an approach should yield higher pyruvate production levels. Too much or too little ATP is not advantageous to cell growth and biosynthetic flux. The results showed that when ATP gene expression was dynamically inhibited by ~25%, the glycolysis flux reached a maximum, with pyruvate production highest and lactic acid accumulation lowest. Tuning the function of biosensors is essential for precise gene expression control. Moreover, the dynamic activation of the cell membrane synthesis pathway is a new regulatory strategy for synthesizing and accumulating lipid‐soluble compounds such as lycopene. To avoid the toxicity of overexpression of the membrane lipid synthesis gene *plsC*, positive‐response biosensors were used to activate *plsC* after cells reached a certain growth point to minimize cytotoxicity and improve product yield. Lycopene production increased 30% and 50‐fold when compared with the non‐*plsC* overexpressing strain and constitutive overexpressing *plsC* strain. Our study demonstrated the effectiveness of promoter design for biosensor development and expands the diversity of regulatory targets for dynamic regulation. The approach should facilitate the broad application of biosensors based on global TFs for precise dynamic control.

## AUTHOR CONTRIBUTIONS

**Yuan Zhu:** Formal analysis (lead); investigation (lead); methodology (lead); writing – original draft (lead). **Huaxiao Gao:** Formal analysis (equal); investigation (equal); methodology (equal). **Jian Zhang:** Methodology (equal); software (equal); validation (equal). **Jingyu Zhao:** Investigation (equal); methodology (equal); validation (equal). **Qingsheng Qi:** Project administration (equal); supervision (equal). **Qian Wang:** Conceptualization (lead); project administration (lead); supervision (lead); writing – original draft (lead); writing – review and editing (lead).

## FUNDING INFORMATION

This work was supported by the National Key R&D Program of China (2019YFA0904900), the Key R&D Program of Shandong Province (2020CXGC010602).

## CONFLICT OF INTEREST

The authors declare no competing interests.

## Supporting information


Appendix S1

## Data Availability

All data produced or analysed for this study are included in the published article or supplementary information files or are available from the corresponding authors upon reasonable request.
