# Supplementary material for: De novo design of the global transcriptional factor Cra‐regulated promoters enables highly sensitive glycolysis flux biosensor for dynamic metabolic control
Source: Microb Biotechnol. 2022 Dec 20;16(3):605–17. doi: 10.1111/1751-7915.14166 (PMC9948231; doi:10.1111/1751-7915.14166)
Supplement: Supplementary file 1 — Appendix S1 [file MBT2-16-605-s001.docx]

**De novo design of the global transcriptional factor Cra-regulated promoters enables highly sensitive glycolysis flux biosensor for dynamic metabolic control**

Yuan Zhu, Huaxiao Gao, Jian Zhang, Jingyu Zhao, Qingsheng Qi, Qian Wang^*^

National Glycoengineering Research Center, State Key Laboratory of Microbial Technology, Shandong University, Qingdao 266237, P. R. China

*Corresponding authors:

Qian Wang Tel: +86532-58631580, E-mail: qiqi20011983[@gmail.com](mailto:qiqi20011983@gmail.com)

Table S1. All the strains used in this study.

| Strains | Relevant characteristic | References |
| --- | --- | --- |
| SW01-SW15 | Strain BW- *pfkA* harboring plasmids pS01-pS15, respectively | This study |
| SW23 | BW25113 *F^-^*, *Δ(araD-araB)567*, *ΔlacZ4787*(::rrnB-3), *λ^-^*, *rph-1*, *Δ(rhaD-rhaB)568*, *hsdR51* | lab stock |
| BW-pyr1 | BW P*_atp_*::Pstm | This study |
| BW-pyr2 | BW P*_atp_*::Pstr | This study |
| BW-pyr3 | BW P*_atp_*::Psto | This study |
| BW-*pfkA* | BW25113*Δ pfkA* | This study |
| SW21 | BW25113 P*_pfkA_*::P23110 | This study |
| SW22 | BW25113 P*_pfkA_*::P23105 | This study |
| SW24 | BW25113 P*_pfkA_*::P23109 | This study |
| SW25 | BW25113 P*_pfkA_*::P23112 | This study |
| BW | BW15113 Δ*acka Δpta ΔpoxB* | This study |
| sly-m1 | DH5α haboring plasmid pLycm1,pEBI5 and pMKD6 | This study |
| sly-m2 | DH5α haboring plasmid pLycm2,pEBI5 and pMKD6 | This study |
| sly-m3 | DH5α haboring plasmid pLycm3,pEBI5 and pMKD6 | This study |
| sly-jh | DH5α haboring plasmid pLyjh,pEBI5 and pMKD6 | This study |
| sly-hh | DH5α haboring plasmid pLyhh,pEBI5 and pMKD6 | This study |
| sly-dxs | DH5α haboring plasmid pPMD,pEBI5 and pMKD6 | This study |
| sly-mep | DH5α haboring plasmid pEBI1 | This study |
| sly01 | DH5α haboring plasmids pMVA,pEBI1 and pMKD1 | This study |
| sly02 | DH5α haboring plasmids pMVA, pEBI1 and pMKD2 | This study |
| sly03 | DH5α haboring plasmids pMVA,pEBI1 and pMKD3 | This study |
| sly04 | DH5α haboring plasmids pMVA, pEBI1 and pMKD4 | This study |
| sly05 | DH5α haboring plasmids pMVA, pEBI1 and pMKD5 | This study |
| sly06 | DH5α haboring plasmids pMVA, pEBI1 and pMKD6 | This study |
| sly07 | DH5α haboring plasmids pMVA, pEBI2 and pMKD1 | This study |
| sly08 | DH5α haboring plasmids pMVA, pEBI2and pMKD2 | This study |
| sly09 | DH5α haboring plasmids pMVA, pEBI2 and pMKD3 | This study |
| sly10 | DH5α haboring plasmids pMVA, pEBI2 and pMKD4 | This study |
| sly11 | DH5α haboring plasmids pMVA, pEBI2 and pMKD5 | This study |
| sly12 | DH5α haboring plasmids pMVA, pEBI2 and pMKD6 | This study |
| sly13 | DH5α haboring plasmids pMVA, pEBI3 and pMKD1 | This study |
| sly14 | DH5α haboring plasmids pMVA, pEBI3 and pMKD2 | This study |
| sly15 | DH5α haboring plasmids pMVA, pEBI3 and pMKD3 | This study |
| sly16 | DH5α haboring plasmids pMVA, pEBI3 and pMKD4 | This study |
| sly17 | DH5α haboring plasmids pMVA, pEBI3 and pMKD5 | This study |
| sly18 | DH5α haboring plasmids pMVA, pEBI3 and pMKD6 | This study |
| sly19 | DH5α haboring plasmids pMVA, pEBI4 and pMKD1 | This study |
| sly20 | DH5α haboring plasmids pMVA, pEBI4 and pMKD2 | This study |
| sly21 | DH5α haboring plasmids pMVA, pEBI4 and pMKD3 | This study |
| sly22 | DH5α haboring plasmids pMVA, pEBI4 and pMKD4 | This study |
| sly23 | DH5α haboring plasmids pMVA, pEBI4 and pMKD5 | This study |
| sly24 | DH5α haboring plasmids pMVA, pEBI4 and pMKD6 | This study |
| sly25 | DH5α haboring plasmids pMVA, pEBI5 and pMKD1 | This study |
| sly26 | DH5α haboring plasmids pMVA, pEBI5 and pMKD2 | This study |
| sly27 | DH5α haboring plasmids pMVA, pEBI5 and pMKD3 | This study |
| sly28 | DH5α haboring plasmids pMVA, pEBI5 and pMKD4 | This study |
| sly29 | DH5α haboring plasmids pMVA, pEBI5 and pMKD5 | This study |
| sly30 | DH5α haboring plasmids pMVA, pEBI5 and pMKD6 | This study |

Table S2. The plasmids used in this study.

| plasmids | Relevant characteristic | Reference |
| --- | --- | --- |
| pEBI1 | pMB1-AMP-P23116-*crtE-crtB-crtI* | This study |
| pEBI2 | pMB1-AMP-P23110-*crtE-crtB-crtI* | This study |
| pEBI3 | pMB1-AMP-P23102-*crtE-crtB-crtI* | This study |
| pEBI4 | pMB1-AMP-P23100-*crtE-crtB-crtI* | This study |
| pEBI5 | pMB1-AMP-Ptrc-*crtE-crtB-crtI* | This study |
| pMKD1 | pCDF -Spc-P23113-*MK-PMK-PMVD-IDI* | This study |
| pMKD2 | pCDF -Spc-P23116-*MK-PMK-PMVD-IDI* | This study |
| pMKD3 | pCDF -Spc-P23110-*MK-PMK-PMVD-IDI* | This study |
| pMKD4 | pCDF -Spc-P23102-*MK*-*PMK*-*PMVD*-*IDI* | This study |
| pMKD5 | pCDF -Spc-P23100-*MK*-*PMK-PMVD*-*IDI* | This study |
| pMKD6 | pCDF -Spc-Ptrc-*MK*-*PMK*-*PMVD*-*IDI* | This study |
| pMVA | p15A-kan-Ptrc-*atoB*-*mvaA*-*mvaS* | ^1^ |
| pLymc1 | p15A-kan- Pm23102- *plsC* | This study |
| pLymc2 | p15A-kan-Pm23100- *plsC* | This study |
| pLymc3 | p15A-kan- Pmtrc - *plsC* | This study |
| pLyjh | p15A-kan-Ptac-*plsC* | This study |
| pLyhh | p15A-kan- P23102-*plsC* | This study |
| pPMD | p15A-kan-Ptrc-*dxs* | This study |
| pS01 | ColEI-Amp-Pm23111-*gfp* | This study |
| pS02 | ColEI-Amp-Pr23111-*gfp* | This study |
| pS03 | ColEI-Amp-Pm23104-*gfp* | This study |
| pS04 | ColEI-Amp-Pr23104-*gfp* | This study |
| pS05 | ColEI-Amp-Pm23102-*gfp* | This study |
| pS06 | ColEI-Amp-Pr23102-*gfp* | This study |
| pS07 | ColEI-Amp-Pm23100-*gfp* | This study |
| pS08 | ColEI-Amp-Pr23100-*gfp* | This study |
| pS09 | ColEI-Amp-Pmtrc-*gfp* | This study |
| pS10 | ColEI-Amp-Prtrc-*gfp* | This study |
| pS11 | ColEI-Amp-Pad-*gfp* | This study |
| pS12 | ColEI-Amp-Paf-*gfp* | This study |
| pS13 | ColEI-Amp-Pstm-*gfp* | This study |
| pS14 | ColEI-Amp-Pstr-*gfp* | This study |
| pS15 | ColEI-Amp-Pun-*gfp* | This study |

Table S3. The primers used in this study.

| **Name** | **Sequence** |
| --- | --- |
| PSM1-F1 | AAGCGGGACCttgacgTTCTTGAAACGTTTCAGCtatagtgctagcTCTAGAGAAAGAGGAGAAATACTAGATGCG |
| PSM1-F2 | CGCTAACCCAACCGGTAACCCCGCTTATTAAAAGCATTCTGTAACAAAGCGGG |
| PS-R | GGTCCCGCTTTGTTACAGAATG |
| PSM3-F1 | CTTGAAACGTTTCAGCtattgtgctagcTCTAGAGAAAGAGGAGAAATACTAGATGCGT |
| PSM3-F2 | TAAAAGCATTCTGTAACAAAGCGGGACCttgacaTTCTTGAAACGTTTCAGCtattgtg |
| PSM5-F1 | CTTGAAACGTTTCAGCtactgtgctagcTCTAGAGAAAGAGGAGAAATACTAGATGCGT |
| PSM5-F2 | TAAAAGCATTCTGTAACAAAGCGGGACCttgacaTTCTTGAAACGTTTCAGCtactgtg |
| PSM7-F1 | CTTGAAACGTTTCAGCtacagtgctagcTCTAGAGAAAGAGGAGAAATACTAGATGCGT |
| PSM7-F2 | TAAAAGCATTCTGTAACAAAGCGGGACCttgacgTTCTTGAAACGTTTCAGCtacagtg |
| PSM9-F1 | ACATTCTTGAAACGTTTCAGCTATAATGTCTAGAGAAAGAGGAGAAATACTAGATGCGT |
| PSM9-F2 | ATTAAAAGCATTCTGTAACAAAGCGGGACCTTGACATTCTTGAAACGTTTCAGCTAT |
| PSM2-F1 | atagtgctagcTTCTTGAAACGTTTCAGCTCTAGAGAAAGAGGAGAAATACTAGATGCG |
| PSM2-F2 | ATTAAAAGCATTCTGTAACAAAGCGGGACCttgacggctagctcagtcctaggtatagtgctagcTTCTTGAAACG |
| PSM4-F1 | attgtgctagcTTCTTGAAACGTTTCAGCTCTAGAGAAAGAGGAGAAATACTAGATGCG |
| PSM4-F2 | ATTAAAAGCATTCTGTAACAAAGCGGGACCttgacagctagctcagtcctaggtattgtgctagcTTCTTGAAACG |
| PSM6-F1 | actgtgctagcTTCTTGAAACGTTTCAGCTCTAGAGAAAGAGGAGAAATACTAGATGCG |
| PSM6-F2 | ATTAAAAGCATTCTGTAACAAAGCGGGACCttgacagctagctcagtcctaggtactgtgctagcTTCTTGAAACG |
| PSM8-F1 | acagtgctagcTTCTTGAAACGTTTCAGCTCTAGAGAAAGAGGAGAAATACTAGATGCG |
| PSM8-F2 | ATTAAAAGCATTCTGTAACAAAGCGGGACCttgacggctagctcagtcctaggtacagtgctagcTTCTTGAAACG |
| PSM10-F1 | TCGTATAATGTTCTTGAAACGTTTCAGCTCTAGAGAAAGAGGAGAAATACTAGATGCGT |
| PSM10-F2 | ATTAAAAGCATTCTGTAACAAAGCGGGACCTTGACAATTAATCATCCGGCTCGTATAATGTTCTTGAAACGTTTCAG |
| PSM11-F1 | TCGTATAATGTTCTTGAAACGTTTCAGCTTCTTGAAACGTTTCAGCTCTAGAGAAAGAGGAGAAATACTAGATGCGT |
| PSM11-F2 | acatgttctttcctgcgttatcccctgattTTGACAATTAATCATCCGGCTCGTATAATGTTCTTGAAACGTTTCAGCT |
| PSM12-F1 | GTTTCAGCTTCTTGAAACGTTTCAGCTTCTTGAAACGTTTCAGCTCTAGAGAAAGAGGAGAAATACTAGATGCGTAAAG |
| PSM12-F2 | CAATTAATCATCCGGCTCGTATAATGTTCTTGAAACGTTTCAGCTTCTTGAAACGTTTCAGCTTCTTGAAACGTTTCAG |
| PSM12-F3 | acatgttctttcctgcgttatcccctgattTTGACAATTAATCATCCGGCTCGTATAATG |
| PSM13-F1 | TTGACAGCTTGAACGATTCACCTAAGATTCTAGAGAAAGAGGAGAAATACTAGATGCGTAAAGG |
| PSM13-F2 | acatgttctttcctgcgttatcccctgattTTGACAGCTTGAACGATTCACCTAAGA |
| PSM14-F1 | TTCGATGAGAGCGATAACGCTTGAACGATTCACCTCTAGAGAAAGAGGAGAAATACTAGATGCGTAAAGGCGAAGAGCT |
| PSM14-F2 | gattAACTGCAAAAATAGTTTGACACCCTAGCCGATAGGCTTTAAGATGTACCCAGTTCGATGAGAGCGATAACGCTTG |
| PSM14-F3 | ctcacatgttctttcctgcgttatcccctgattAACTGCAAAAATAGTTTGACACCC |
| PSM15-F1 | ACttttgctggccttttgGCTTGAACGATTCACCTCTAGAGAAAGAGGAGAAATACTAGATGCGTAAAGGCGAAGAGCT |
| PSM15-F2 | AATAGTTTGACACCCTAGCCGATAGGCTTTAAGATGTACCCAGTTCGATGAGAGCGATAACttttgctggccttttgGC |
| PSM15-F3 | acatgttctttcctgcgttatcccctgattAACTGCAAAAATAGTTTGACACCCTAGCCG |
| PE-bone-F | GCAGGTTTGATGCTGGAGGATCTGATATGAAAAGCTTTCTAGAACAAAAACTCATCTCA |
| PE-bone-R | cataatccctaggactgagctagccatcagCAGCTCATTTCAGAATATTTGCCAG |
| crtE-F1 | TGAGCTGctgatggctagctcagtcctagggattatgctagcTGTGGAATTGTGAGCGGATAACAATTTC |
| crtE-F2 | ATCATAACGGTTCTGGCAAATATTCTGAAATGAGCTGctgatggctagct |
| crtE-R | TACCTCCTTTAGGGATAATAGCTGTTTGCCTTAACTGACGGCAGCGAGTT |
| crtB-F1 | GCCGTCAGTTAAGGCAAACAGCTATTATCCCTAAAGGAGGTACTAGATGAATAATCCGTCGTTACTCAATCATGC |
| crtB-F2 | TTATTCAGGCCTGGTTTGACAAAAAACTCGCTGCCGTCAGTTAAGGCAAACAG |
| crtB-R | TACCTCCTTTAGGGATAATAGCTGTTTGCCCTAGAGCGGGCGCTGCCAGA |
| crtI-F1 | AGCGCCCGCTCTAGGGCAAACAGCTATTATCCCTAAAGGAGGTACTAGATGAAACCAACTACGGTAATTGGTG |
| crtI-F2 | CCGCCCTGCGCATCTCTGGCAGCGCCCGCTCTAGGGCAAA |
| crtI-R | CTGAGATGAGTTTTTGTTCTAGAAAGCTTTTCATATCAGATCCTCCAGCATCA |
| PK-bone-F | GAAAATGACAGGCAAATTCATAGAATGCTATAAGGGCCCTGTCATCTCTTCGTTA |
| PK-bone-R | AgctagcataatccctaggactgagctagccatcagTGCACCTTCATGGTGGTCAGTGCG |
| MK-F1 | gctcagtcctagggattatgctagcTTGTGAGCGGATAACAAAAGGAGGTAAAAAAACATGGTATCCTGTTCTGCGCCG |
| MK-F2 | ATCAGCAGGACGCACTGACCACCATGAAGGTGCActgatggctagctcagtcctagggattatgct |
| MK-R | TCCTTAAGGGTGCAGGCCTATCGCAAATTAGCTTAATCTACTTTCAGACCTTGCTC |
| PMK-F1 | ttgacagctagctcagtcctagggactatgctagcTGTGGAATTGTGAGCGGATAACAA |
| PMK-F2 | AAACCGACCGAGCAAGGTCTGAAAGTAGATTAAGCTAATTTGCGATAGGCCTGCACC |
| PMK-R | GGCGAATTCTGCATGCAGCTACCTTAAGTTATTTATCAAGATAAGTTTCCGGATCTT |
| MVD-F1 | CTTAAGGTAGCTGCATGCAGAATTCGCCCTTAAGGAGGAAAAAAAAATGACCGTTTACACAGCATCC |
| MVD-F2 | AAAAGATCCGGAAACTTATCTTGATAAATAACTTAAGGTAGCTGCATGCAGAA |
| MVD-R | ACCTCCTAAGGGCGATGCAGCGAATTGATCTTATTCCTTTGGTAGACCAGTCTTT |
| IDI-F1 | GATCAATTCGCTGCATCGCCCTTAGGAGGTAAAAAAAAATGACTGCCGACAACAATAGTATG |
| IDI-F2 | GACGCAAAGACTGGTCTACCAAAGGAATAAGATCAATTCGCTGCATCGCCC |
| IDI-R | AACATTAATAACGAAGAGATGACAGGGCCCTTATAGCATTCTATGAATTTGCCTGTCA |
| PE2-F1 | tttacggctagctcagtcctaggtacaatgctagcTGTGGAATTGTGAGCGGATAACAA |
| PE2-F2 | CGGTTCTGGCAAATATTCTGAAATGAGCTGtttacggctagctcagtcctaggt |
| PE2-R | CAGCTCATTTCAGAATATTTGCCAG |
| PE3-F1 | ttgacagctagctcagtcctaggtactgtgctagcTGTGGAATTGTGAGCGGATAACAA |
| PE3-F2 | CGGTTCTGGCAAATATTCTGAAATGAGCTGttgacagctagctcagtcctaggt |
| PE4-F1 | ttgacggctagctcagtcctaggtacagtgctagcTGTGGAATTGTGAGCGGATAACAA |
| PE4-F2 | CGGTTCTGGCAAATATTCTGAAATGAGCTGttgacggctagctcagtcctaggt |
| PE5-F1 | GAGCTGTTGACAATTAATCATCCGGCTCGTATAATGTGTGGAATTGTGAGCGGATAACAAT |
| PE5-F2 | TAACGGTTCTGGCAAATATTCTGAAATGAGCTGTTGACAATTAATCATCCGG |
| PK2-F1 | ctagggactatgctagcTTGTGAGCGGATAACAAAAGGAGGTAAAAAAACATGGTATCC |
| PK2-F2 | GCAGGACGCACTGACCACCATGAAGGTGCAttgacagctagctcagtcctagggactatgctagcTTGTGA |
| PK2-R | TGCACCTTCATGGTGGTCAGTG |
| PK3-F1 | ctaggtacaatgctagcTTGTGAGCGGATAACAAAAGGAGGTAAAAAAACATGGTATCC |
| PK3-F2 | GCAGGACGCACTGACCACCATGAAGGTGCAtttacggctagctcagtcctaggtacaatgctagcTTGTGAGC |
| PK4-F1 | ctaggtactgtgctagcTTGTGAGCGGATAACAAAAGGAGGTAAAAAAACATGGTATCC |
| PK4-F2 | GCAGGACGCACTGACCACCATGAAGGTGCAttgacagctagctcagtcctaggtactgtgctagcTTGTGAG |
| PK5-F1 | ctaggtacagtgctagcTTGTGAGCGGATAACAAAAGGAGGTAAAAAAACATGGTATCC |
| PK5-F2 | GCAGGACGCACTGACCACCATGAAGGTGCAttgacggctagctcagtcctaggtacagtgctagcTTGTGAG |
| PK6-F1 | AGGTGCATTGACAATTAATCATCCGGCTCGTATAATGTTGTGAGCGGATAACAAAAGGAGGTAAAAA |
| PK6-F1 | CATCAGCAGGACGCACTGACCACCATGAAGGTGCATTGACAATTAATCATCCGG |
| dxs-F1 | CATCGGCTCGTATAATGTCTAGAGAAAGAGGAGAAATACTAGatgAGTTTTGATATTGCCAAATACCCG |
| dxs-F2 | gtggtatggctgtgcaggtcgtaaatcactTTGACAATTAATCATCGGCTCGTATAATGTCTAGAG |
| dxs-R | ggagtgcgccttgagcgacacgaattatgcttaTGCCAGCCAGGCCTTGAT |
| PMVA-F | gcataattcgtgtcgctcaagg |
| PMVA-R | agtgatttacgacctgcacagc |

Table S4: The sequence of promoter used in this study.

| Name | Sequence |
| --- | --- |
| P_23100_ | ttgacggctagctcagtcctaggtacagtgctagc |
| P_23101_ | tttacagctagctcagtcctaggtattatgctagc |
| P_23102_ | ttgacagctagctcagtcctaggtactgtgctagc |
| P_23103_ | ctgatagctagctcagtcctagggattatgctagc |
| P_23104_ | ttgacagctagctcagtcctaggtattgtgctagc |
| P_23105_ | tttacggctagctcagtcctaggtactatgctagc |
| P_23106_ | tttacggctagctcagtcctaggtatagtgctagc |
| P_23107_ | tttacggctagctcagccctaggtattatgctagc |
| P_23108_ | ctgacagctagctcagtcctaggtataatgctagc |
| P_23109_ | tttacagctagctcagtcctagggactgtgctagc |
| P_23110_ | tttacggctagctcagtcctaggtacaatgctagc |
| P_23111_ | ttgacggctagctcagtcctaggtatagtgctagc |
| P_23112_ | ctgatagctagctcagtcctagggattatgctagc |
| P_23113_ | ctgatggctagctcagtcctagggattatgctagc |
| P_23114_ | tttatggctagctcagtcctaggtacaatgctagc |
| P_23115_ | tttatagctagctcagcccttggtacaatgctagc |
| P_23116_ | ttgacagctagctcagtcctagggactatgctagc |
| P_23117_ | ttgacagctagctcagtcctagggattgtgctagc |
| P_23118_ | ttgacggctagctcagtcctaggtattgtgctagc |
| P_trc_ | ttgacaattaatcatccggctcgtataatg |
| P_m23111_ | ttgacgttcttgaaacgtttcagctatagtgctagc |
| P_r23111_ | ttgacggctagctcagtcctaggtatagtgctagcttcttgaaacgtttcagc |
| P_m23104_ | ttgacattcttgaaacgtttcagctattgtgctagc |
| P_r23104_ | ttgacagctagctcagtcctaggtattgtgctagcttcttgaaacgtttcagc |
| P_m23102_ | ttgacattcttgaaacgtttcagctactgtgctagc |
| P_r23102_ | ttgacagctagctcagtcctaggtactgtgctagcttcttgaaacgtttcagc |
| P_m23100_ | ttgacgttcttgaaacgtttcagctacagtgctagc |
| P_r23100_ | ttgacggctagctcagtcctaggtacagtgctagcttcttgaaacgtttcagc |
| P_mtrc_ | ttgacattcttgaaacgtttcagctataatg |
| P_rtrc_ | ttgacaattaatcatccggctcgtataatgttcttgaaacgtttcagc |
| P_ad_ | ttgacaattaatcatccggctcgtataatgttcttgaaacgtttcagcttcttgaaacgtttcagc |
| P_af_ | ttgacaattaatcatccggctcgtataatgttcttgaaacgtttcagcttcttgaaacgtttcagcttcttgaaacgtttcagcttcttgaaacgtttcagc |
| P_stm_ | ttgacagcttgaacgattcacctaagat |
| P_str_ | aactgcaaaaatagtttgacaccctagccgataggctttaagatgtacccagttcgatgagagcgataacgcttgaacgattcacc |
| P_un_ | Aactgcaaaaatagtttgacaccctagccgataggctttaagatgtacccagttcgatgagagcgataaccagcaacgcggcctttgcttgaacgattcacc |

Table S5: The sequence of TFBS used in this study.

| Name | Sequence |
| --- | --- |
| O_ppsA_ | ggtgaatcgttcaagc |
| O_fruB_ | gctgaaacgtttcaagaa |

**Fig S1:**

**Supplementary Fig 1. Characterization of the promoters** **P_23111_, P_23104_, P_23102_, P_23100_, P_trc_. The strength of these five promoters ranking was P_trc_ > P_23100_> P_23102_> P_23104_> P_23111._**

**Supplementary References**

1. Wang, Q.; Xu, J.; Sun, Z.; Luan, Y.; Li, Y.; Wang, J.; Liang, Q.; Qi, Q., Engineering an in vivo EP-bifido pathway in Escherichia coli for high-yield acetyl-CoA generation with low CO2 emission. *Metab Eng* **2019,** *51*, 79-87.
